# High-frequency audiometry in young and older adults when conventional audiometry is normal

**DOI:** 10.1016/S1808-8694(15)31024-7

**Published:** 2015-10-19

**Authors:** Isabella Monteiro de Castro Silva, Maria Ângela Guimarães Feitosa

**Affiliations:** a Master's degree, Adjunct Professor at the Centro Universitário Planalto do Distrito Federal.; b PhD on Psychology, Professor at the University of Brasilia. UNIPLAN - Centro Universitário Planalto do Distrito Federal - UnB - University of Brasilia

**Keywords:** High frequency audiometry, Presbiacusis, Auditory aging, Hearing sensitivity

## Abstract

High-frequency audiometry can detect early changes in auditory sensitivity resulting from processes such as aging. Nonetheless its use is still limited, and additional studies are required to establish its use, particularly among older adults.

**Aim:**

To compare pure tone thresholds for frequencies from 250 Hz to 16 kHz in young and older adults, with or without audiologic complaints.

**Method:**

Pure tone sensitivity to 250 Hz to 16 kHz was assessed with an AC-40 audiometer in 64 adults, evenly distributed in young (25 to 35 years-old) and older (45 to 55 years-old) adults of both sexes. This is a cross-sectional study.

**Results:**

Although all participants presented normal audiometry in frequencies from 250 Hz to 8 kHz, according to clinical parameters, older adults had significantly higher thresholds compared to young adults, according to statistical parameters, with greater significance in higher frequencies (8 to 16 kHz). Presence or absence of clinical complaints did not distinguish thresholds.

**Conclusions:**

The process of auditory aging, including loss of sensitivity to higher frequencies, can be detected at earlier ages than those usually investigated. High frequency audiometry is an important instrument to distinguish auditory sensitivity in young and older adults, even for those considered as audiologically normal.

## INTRODUCTION

### Age-Associated Hearing Loss

Presbyacusis, or age-associated hearing loss, refers to aging changes that take place in the auditory system such as changes in the anatomy and function of the external and middle ear. These alterations also involve sensory, nerve, vascular striae, and Corti organ support cells, and changes in the plasticity of central nervous processing which include the cochlear nucleus, the superior olivary nucleus, the lateral lemniscus, the inferior colliculus, and the medial geniculate body. Such progressive degeneration involves both ears symmetrically, initially affecting frequencies over 2,000Hz,^1^, ^2^, ^3^, ^4^ and take place earlier in men compared to women.^4^, ^5^

Certain environmental factors may have an influence on the degree of hearing loss at high frequencies, including the diet, contact or intake of chemical agents and medical drugs, and noise exposure.^6^, ^7^, ^8^

A significant methodological challenge in studying presbyacusis is to filter environmental variables and to collect the pure phenomenon.^7^, ^9^, ^10^ One needs to include the largest possible number of risk factors for hearing loss in order to clarify the auditory sensorial aging process. This includes interviews on life habits, health and hearing, and a detailed otological assessment. Knowing the variables in a study population allows an assessment of the part played by these variables in the aging process.

Hearing loss leads to social isolation in aging persons.^1^, ^11^ This includes losses in speech recognition in noisy social environments.^6^ Research in this area has attempted to describe the aging process as related to hearing, and has concentrated efforts on the study of audiometric thresholds, auditory processing and speech perception. These studies have contributed to the early detection of sensory or neural changes, which may interrupt or minimize the social exclusion effect, consequently improving the quality of life, autonomy and independence of people with hearing loss.^12^, ^13^, ^14^ An early and adequate diagnosis has an important role in defining the medical strategy, such as adapting sound amplification devices and rehabilitation procedures for auditory function in the elderly.

One of the strategies used to study age-associated hearing loss is to compare pure-tone thresholds in young and old people. However, not only sensory factors, but neural and cognitive aspects are also involved in people over 60 years, which complicates the evaluation of the effect of each variable on hearing and its aging process. Therefore, it is useful to study a younger population to precisely characterize the beginnings of age-related hearing loss.

### Assessment Procedures

Various procedures may be used to assess hearing. These include physiological tests - otoacoustic emissions, auditory evoked potentials, immitance testing, behavioral or psychoacoustic tests - pure-tone audiometry and logoaudiometry. Physiological tests assess the response of the auditory system (sensory cells and the auditory neural pathways), objectively capturing the responses; these tests are highly specific and sensitive, with little response variability.^15^, ^16^ However, they do not assess hearing, as individual responses are not taken into account.

Behavioral tests aim to assess the peripheral and central auditory system, showing the effective response based on what the subjects hears. Pure-tone audiometry is a behavioral, psychoacoustic standardized procedure that describes auditory sensitivity. Conventional audiometry testes frequencies between 250Hz and 8,000Hz, and high-frequency audiometry tests frequencies between 10,000Hz and 20,000Hz. Normal standards for conventional audiometry are based on average thresholds at 500Hz, 1 and 2kHz below 25 dB.^17^ High-frequency audiometry (over 8kHz) is useful to measure cochlear function, diagnosing sensorial injury earlier than conventional audiometry.^18^ Furthermore, high-frequency audiometry is a sensitive tool to differentiate groups of individuals classified by conventional audiometry as audiologically normal, in which one may have no hearing complaints (control group) and the other may present difficulties of speech recognition in noisy environments (test group).^19^ Test group participants have been classified according to a range of clinical tests as having Obscure Auditory Dysfunction (OAD). OAD individuals have a significant threshold increase, which means that high-frequency audiometry may be an ancillary test for clinical investigation.^19^

Normative data on absolute thresholds at high frequencies according to age were collected in a sample of 240 subjects aged between 10 and 59 years that had thresholds below 25 dBNA at conventional frequencies.^20^ Increased pure-tone thresholds, in dBNPS, occurred more rapidly as a function of age for frequencies between 13 and 17kHz. Another study of the absolute threshold (dBNA) at high frequencies (10; 12.5; 14 and 16kHz) was also done in Brazil with subjects aged between 4 and 60 years, having auditory thresholds below 25 dBNA at frequencies of 250Hz to 8kHz.^21^ According to the authors, thresholds were higher at 16kHz, 12.5kHz and 14kHz. There was a critical aging effect at 16kHz, as differences were statistically significant in successive decades (30 to 60 years); each decade from 30 years onwards shows a considerable change in the pure-tone threshold at this frequency. Notwithstanding these studies, high-frequency thresholds have not been standardized due to the different sound emission modes (dBNA and dBNPS) and the wide inter-subject variability, which complicates comparisons between findings in different studies.

Our study originated from questions raised about the use of high-frequency audiometry to differentiate individuals with clinically normal conventional audiometry and with or with no hearing complaints. We also sought to characterize age-associated hearing loss from age 30 onwards. The study, therefore, was aimed at detecting pure-tone frequencies between 250Hz and 16kHz, and comparing conventional auditory and high-frequency thresholds. Data analysis was made to compare the thresholds of young adults (25-35 years) with no risk of presbyacusis with a group of middle-aged adults (45-55 years) with initial age-associated hearing loss. We wished to observe variables such as age, gender and hearing complaints, using conventional and high-frequency audiometry.

## CASES AND METHOD

### Participants

This study was approved by the Research Ethics Committee of the Brasilia University Sciences Faculty (protocol 003/2003) and data collection took place between February and August 2003. There were 64 participants in the study, all of which were patients of the Clinical Otorhinolaryngology Audiology Unit in Brasilia-FD. Participants were divided into four groups organized by age and gender (Table 1). Each group was subdivided in two according to the presence or not of auditory or hearing complaints; these had been investigated during routine clinical history taking. We were left with 32 participants with complaints and 32 participants with no complaints. All participants had at least completed secondary schooling as a general inclusion criterion; the intention of this was to control for cultural and intellectual variables. Young adults (25-35 years) had professional or recreational activities that required intellectual capability, and older adults (45-55 years) were recruited among persons that had professional activities. The final inclusion of volunteers as participants was confirmed based on the result of a set of procedures described below.

**Table 1:** Age of participants (average and variation in years) according to gender and condition of complaint.

Group	Condition of complaint	
	No complaint	Complaint
Young Female	29.1 (25-33)	27.5 (25-30)
Young Male	29.8 (25-35)	31.7 (27-35)
Older Female	50.5 (49-53)	49.6 (45-54)
Older Male	50.2 (46-55)	48.3 (45-52)

Survey of the general and auditory health and exposure to risk factors for hearing loss

Open questions in an interview setting and semi-structured questionnaires were used to investigate the presence or not of auditory or hearing complaints. There were both yes/no and open answers. Participants were also given the opportunity of defining their auditory complaints in free writing. Information was obtained about the general health, auditory health, and family, social and occupational risk factors for hearing loss. Based on this information, participants were divided into groups with or without auditory complaints. Signing the consent form and filling in the questionnaire took, on average, 30 minutes.

Participants had no other prenatal, perinatal and postnatal clinical findings that could exclude them from the sample; our data did not find indicators suggesting hearing loss. The risk factors presented by the participants are shown on Table 2. The questionnaire was also useful to characterize auditory and non-auditory complaints for each participant. Auditory complaints uncovered in the questionnaire are shown on Table 3, and non-auditory complaints are shown on Table 4.

**Table 2:** Distribution of risk factors for hearing loss in all groups of participants.

Risk Factors					Groups				
	JFS*	JFC*	JMS*	JMC*	VFS*	VFC*	VMS*	VMC*	Total
Clinical events at birth	3	3	2	2	0	3	1	0	14
Family history of loss	3	2	3	4	2	7	4	2	27
Occupational noise	0	4	2	3	1	0	2	1	13
Noise during leisure	1	1	3	3	1	2	2	3	16
Noise - occupational and leisure	1	1	2	1	1	2	1	0	9
Smoking	1	0	1	2	2	0	1	3	10
General health									
Hypothyroidism	1	1	0	0	1	0	0	0	3
Respiratory problems	1	3	0	0	0	1	0	0	5
Hypertension	0	0	0	0	0	1	0	0	1
Leprosy	0	0	0	0	0	1	0	0	1
Hearing health									
Otitis in infancy	3	3	1	2	0	2	1	2	14

Total	14	18	14	17	8	19	12	11	113

* JFS - young females with no auditory complaints; JFC - young females with auditory complaints; JMS - young males with no auditory complaints; JMC - young males with auditory complaints; VFS - older females with no auditory complaints; VFC - older females with auditory complaints; VMS - older males with no auditory complaints; VMC - older males with auditory complaints.

**Table 3:** Distribution of auditory complaints presented in all groups of participants.

Auditory complaints					Groups				
	JFS*	JFC*	JMS*	JMC*	VFS*	VFC*	VMS*	VMC*	Total
Speech recognition	0	1	0	3	0	4	0	2	10
Tinnitus	0	6	0	5	0	6	0	4	21
Hearing loss	0	1	0	3	0	0	0	3	7
Hypersensitivity	0	1	0	1	0	0	0	0	2

Total	0	9	0	12	0	10	0	9	40

* JFS - young females with no auditory complaints; JFC - young females with auditory complaints; JMS - young males with no auditory complaints; JMC - young males with auditory complaints; VFS - older females with no auditory complaints; VFC - older females with auditory complaints; VMS - older males with no auditory complaints; VMC - older males with auditory complaints.

**Table 4:** Distribution of non-auditory complaints presented in all groups of participants.

Non-Auditory Complaints	Groups								
	JFS	JFC	JMS	JMC	VFS	VFC	VMS	VMC	Total
Vertigo	0	2	1	0	2	5	1	0	11
Pain	0	2	0	0	1	2	0	1	6
Pruritus	2	0	0	1	2	0	0	1	6
Pressure	0	1	0	0	0	0	0	1	2

Total	2	5	1	1	5	7	1	3	25

^*^ JFS - young females with no auditory complaints; JFC - young females with auditory complaints; JMS - young males with no auditory complaints; JMC - young males with auditory complaints; VFS - older females with no auditory complaints; VFC - older females with auditory complaints; VMS - older males with no auditory complaints; VMC - older males with auditory complaints.

### Timpanometry

All participants underwent tympanometry prior to threshold assessment procedures using the immitance testing device AZ 26 - Interacoustics. This was to assure the integrity of the external and middle ear. According to the sample selection criteria, all participants had a type A curve, suggesting adequate function of the tympanoossicular system.

### Pure-tone threshold assessment

Pure-tone audiometry was done in a answer button-equipped acoustic chamber at frequencies of 250, 500, 1000, 2000, 3000, 4000, 6000 and 8000Hz. We used a two-channel audiometer (AC 40 Clinical Audiometer - Interacoustics) to obtain information about hearing at traditionally tested frequencies in medical audiology. High-frequency thresholds (10, 12.5 and 16kHz) were also checked to obtain information about the basal portion of the cochlea. Frequencies over 16kHz were not assessed due to equipment limits. A TDH 39standard earphone was used to assess the tone threshold between 0.25 and 8kHz. A KOSS HV/PRO high-frequency earphone was used to assess frequencies over 8kHz.

We adopted the automatic pure-tone test procedure (Hughson Westlake) to assess the threshold for two correct answers in three attempts. The intensity was reduced in 10 dB steps after each detection response; intensity was raised by 5 dB after each lack of response. Participants were asked to hear tones at various frequencies and intensities and to press the button each time the tone was audible, however minimal. All participants had pure-tone thresholds below 25 dBNA at frequencies between 500Hz to 2kHz, which is considered within normal limits.

## RESULTS

Audiometry results were analyzed using the arithmetic mean to investigate the general trend for each of the eight subgroups. As some individuals did not respond at maximal audiometer acoustic output intensities, a threshold value was given for an absent response 5 dB above the maximum output intensity. Thus, at 12.5kHz, which has a maximum 60 dBNA output, the value 65 dBNA was attributed as a threshold for cases in which there was no response. At 16kHz, which has a maximum output of 40 dBNA, 45 dBNA was considered as the threshold for individuals with no response up to this limit. This approach was not required for other frequencies, as all thresholds were below the maximal output.

Multivariate analysis using the software SPSS 11.5 was done to test age group effects (young adults and older adults), gender effects and auditory condition of complaint effects (with or with no complaint) on thresholds for each tested frequency (Table 5). Pure-tone thresholds were fed into the software as dependent variables. The age group, gender and condition of complaint were inserted into the software as factors. The condition of complaint had no significant effect over thresholds at any frequency we studied, suggesting that the groups with and with no complaints had similar thresholds. These two groups then became a single group, eliminating the factor complaint from data analysis.

**Table 5:** Results of the threshold multivariate analysis according to the age group.

Frequency (kHz)	F	g.l.	p
0.25	14.6	1	< 0.001
0.5	7.0	1	0.010
1	9.1	1	0.004
2	9.7	1	0.003
3	8.5	1	0.005
4	9.4	1	0.003
6	6.9	1	0.011
8	13.7	1	< 0.001
10	39.7	1	< 0.001
12.5	117.1	1	< 0.001
16	63.0	1	< 0.001

**Fig.1:**
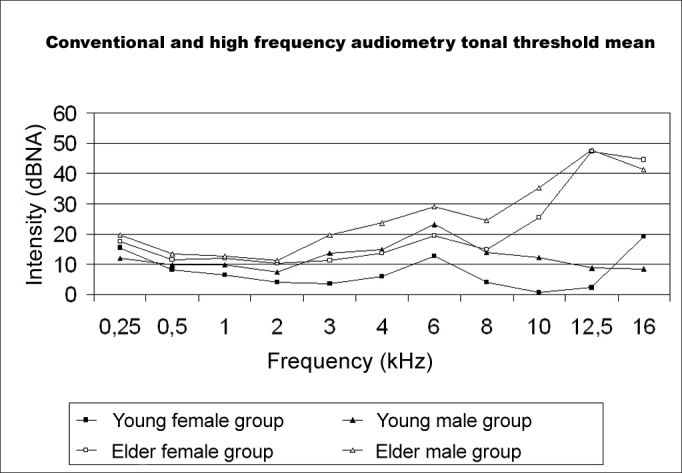
Average pure-tone threshold for young and older male and female adults in conventional and high-frequency audiometry.

Figure 1 shows pure-tone threshold averages, including significant factors such as the age group and gender. Results were organized in four groups - young female, young male, older female and older male.

In the younger groups (25 to 35 years) both men and women had thresholds below 25 dBNA at all tested frequencies (250Hz to 16kHz). Sensitivity was higher at frequencies between 8 and 12.5kHz, and lower at 16kHz. In the older groups (45 to 55 years), thresholds were below 25 dBNA at frequencies between 250Hz and 8kHz except at 6kHz. Over 10kHz, thresholds increased up to 60 dBNA at 12.5kHz and 40 dBNA at 16kHz. Although thresholds were similar between 250Hz and 4kHz (below 25 dBNA), thresholds in the older groups were significantly higher compared to the younger groups at all frequencies (Table 5). Gender was a factor that separated age groups at frequencies between 3kHz and 10kHz, but had no significant effect at other frequencies. Women had statistically lower thresholds than men at the abovementioned frequencies. Interactions between age group and gender were not significant, suggesting an independent effect of the factors age and gender on thresholds at each frequency.

The threshold difference between the younger and older groups at 12.5kHz was lower at 16kHz, although still significant. At 12.5kHz, thresholds in the younger groups were below 10 dBNA, whereas thresholds of older adults were over 45 dBNA. At 16kHz, all of the older groups converged to a 45 dB threshold, while younger groups had more widely dispersed thresholds, varying from -5 dBNA to 25 dBNA, similar to the curves shown on Figure 1. This difference-reduction effect between older and younger groups at 16kHz may be an artifact related to the limits of the audiometer used in this study. In order to adequately interpret this information, we should understand that all groups had thresholds above the audiometer limit, that is, over 40 dBNA at 16kHz. Therefore, the difference between the thresholds of older and younger groups at 16kHz should be seen as probably higher than what is shown on Figure 1.

## DISCUSSION

### Age Effect

Notwithstanding the small sample size, there was a significant age group effect on thresholds, with reduced auditory sensitivity and increased thresholds at all frequencies in the older groups. Although clinically classified as audiologically normal, the analysis separated younger and older individuals by indicating statistically different thresholds for each (Table 5).

Sensitivity assessments at higher frequencies had been done previously, showing increased thresholds in parallel with increasing age.^21^, ^20^ Another paper^22^ revealed higher thresholds in elderly individuals compared to younger individuals. By masking both groups before testing again for thresholds, elderly individuals were again seen to have higher thresholds compared to younger individuals, although there was a threshold rise in the younger group.

Studies of young individuals with no hearing loss^10^ showed significant threshold (dBNPS) increases at 12kHz and above, and reduced thresholds at 20kHz. Auditory sensitivity in dBNA improved as a function of increased frequency,^23^ partially in disagreement with literature results.^10^ Results in this study showed better sensitivity at higher frequencies in young individuals, with a reduction at 16kHz in women. Literature^21^ shows higher thresholds at 16, 12.5 and 14kHz, in this order. Other authors point to a lower threshold at 12kHz.^10^ A higher threshold was seen at 12.5kHz (60 dBNA) compared to 16kHz, different from other studies;^21^ this is probably due to the maximum output limit of the audiometer (60 dBNA at 12.5kHz and 40 dBNA at 16kHz). However, the importance of the 12.5kHz frequency in separating groups by age, seen in our study, is consistent with data presented in other papers.^10^

Age-associated hearing loss initially affects higher frequencies.^2^, ^5^, ^20^, ^24^, ^25^ In this study we show groups of older adults (45 to 55 years) with significantly worse pure-tone sensitivity compared to younger adults (25 to 35 years). Some papers report ages close to 40 years,^18^, ^20^, ^25^ while another study^5^ detected reduced auditory sensitivity in men aged 30 years. Following normal sensitivity criteria, all participants in our study had auditory thresholds within normal limits. High-frequency sensitivity, however, was reduced in the 45 to 55 year age group. These findings corroborate literature findings and suggest that high-frequency audiometry may be used clinically for the early diagnosis of age-associated hearing loss.

### Gender Effect

Gender had a statistically significant effect at frequencies between 3kHz and 10kHz. Women had lower thresholds compared to men, which coincides with a previous study done only with conventional frequencies (up to 8kHz).^18^ Men had higher thresholds than women at frequencies above 3kHz. Literature shows that the beginning of the auditory sensitivity loss process has little effect on lower frequencies, affecting mostly higher frequencies in men.^5^, ^24^ Age-associated hearing loss is slower in women, affecting all frequencies uniformly, resulting in horizontal audiometric curves.^24^

Statistically significant differences in pure-tone sensitivity at 250 to 8,000Hz were seen between men and women.^26^ Over 1,000Hz, thresholds in men increased at twice the speed compared to women. According to the authors, these findings^26^ may be explained by men being more exposed to noise than women, which causes injury before age-associated hearing losses affect sensorial cells. A further study^18^ tried to eliminate risk factors from its sample, including noise exposure, and assessed pure-tone sensitivity at 500 to 8,000Hz in two age groups. The age factor was seen at all frequencies, and gender was important at frequencies over 3,000Hz. Our analysis of the semi-structured questionnaire filled in by participants in our sample showed that the noise factor was present in the reports of 15 out of 32 participating women (46%) and in the reports of 23 out of 32 participating men (72%). This is consistent with literature findings that report increased male exposure to noise compared to women. Our results are similar to literature results that tried to minimize the noise effect in their samples.^18^

### Complaint Effect

The presence or absence of an auditory complaint had no effect on conventional or high-frequency pure-tone thresholds. It should be noted that the complaint investigation survey initially contained open questions, followed by a questionnaire with closed items and free spaces for participant reports. A detailed survey was done^19^ of speech recognition complaints using a test battery specifically developed to detect organic and functional components of OAD, a condition of normal sensitivity associated with speech recognition changes in noisy environments. The questionnaire we used was elaborated to identify risk factors for hearing loss and auditory complaints in the lowest possible time to avoid second participant visits for research purposes. Sensitivity may have been reduced by grouping questions directly or indirectly related to various aspects of hearing, such as auditory complaints, risk factors and cognition. A test battery using specific tools might have detected each of these aspects with greater precision. Furthermore, the fact that participants themselves filled in the questionnaires may have reduced precision, and consequently the complaint effect.

## CONCLUSION

The most significant results in our study were:

(a) Pure-tone thresholds for tested frequencies in conventional audiometry (250Hz to 8kHz) were increased in the older group compared to the younger group, although all participants had been classified as audiologically normal. These differences were statistically significant. At higher frequencies (10 to 16kHz), the tendency for increased thresholds in the older groups was increased still further.
(b) Pure-tone thresholds in females were statistically lower at higher frequencies (3 to 10kHz) compared to males, suggesting that aging begins earlier in men.
(c) The complaint effect was not observed at the thresholds tested in this study.
(d) High-frequency audiometry may be used in audiology clinics as a routine procedure with advantages in diagnosis. In our sample this procedure was more sensitive to detect the progression of hearing threshold increases in adults.
